# 

**Published:** 1896-03-07

**Authors:** 


					-1"    " ABSOLUTELY PURE, therefore BEST. March 7th, 1898.
CADBURYS ?, CADBURYS
y? cocoa cocoa
PUrity " PreSCnt ^ ^ ^ NO ALKALIES USED.
No. 493. Vol. XIX.
Saturday,
March 7th, 1896.
WITH EXTRA NURSING SUPPLEMENT.
[Registered at the General Post Office as a Newspaper for Monthly Parts, 6d.; post free, 8d.
transmission in the United Kingdom and Abroad,] Ditto per Annnm, 8s.6d., post free.

				

